# Beyond recognition: Refining the assessment of public knowledge and risk perception in dementia prevention

**DOI:** 10.1016/j.tjpad.2026.100602

**Published:** 2026-05-20

**Authors:** Zhiyan Xie, Jun Su, Tao Liao

**Affiliations:** a Department of Geriatric Medicine, Neijiang Hospital of Traditional Chinese Medicine, Neijiang, Sichuan 641000, China; b Department of Cardiopulmonary Diseases, Neijiang Hospital of Traditional Chinese Medicine, Neijiang, Sichuan 641000, China

To the Editor,

We read with great interest the systematic review and meta-analysis by Sambou et al. [1], which provides a timely and comprehensive overview of public knowledge and perception of dementia risk and protective factors. By synthesizing evidence from 155 studies involving 164,644 participants across 41 countries, the authors convincingly show that public awareness of many established modifiable dementia risk factors remains limited, particularly for obesity, air pollution, education, and cardiometabolic factors. The distinction between recognition and recall is especially valuable, as it suggests that even apparently moderate awareness may overestimate the knowledge that people can actively use in daily health decisions.

Nevertheless, three additional issues may deserve consideration. First, knowledge of a risk factor does not necessarily indicate actionable understanding. A respondent may recognize that physical inactivity, hypertension, or hearing loss is related to dementia, but may not understand why the association matters, when intervention is most effective, or what specific action should be taken. This distinction is important because dementia prevention depends not only on awareness, but also on mechanistic understanding, perceived personal relevance, and practical self-efficacy [2]. Future surveys could therefore move beyond binary recognition by assessing whether people know the life-course timing, intervention pathway, and realistic preventive actions linked to each risk factor.

Second, the public health meaning of low awareness may differ substantially across risk factors [3]. Physical activity, smoking, and alcohol use are largely individual behavioral targets; hypertension, diabetes, obesity, and hearing loss require clinical screening and long-term management; education, air pollution, and neighborhood environments are predominantly structural or policy-level determinants [4]. Interpreting all knowledge gaps as a need for public education may therefore be incomplete. Future work may benefit from classifying dementia risk factors according to their actionability and intervention pathway, thereby distinguishing messages aimed at individual behavior change from those requiring health-system integration or policy advocacy.

Third, dementia risk perception may conflate rational risk appraisal with emotional fear and disease stigma. Worry or fear about dementia may arise from caregiving experience, anticipated loss of autonomy, or social stigma rather than calibrated understanding of personal risk [5]. Conversely, low perceived risk may not always reflect ignorance, but may reflect confidence in existing preventive behaviors [6]. Future studies should separately measure perceived susceptibility, objective risk calibration, fear, stigma, perceived preventability, and self-efficacy.

In conclusion, Sambou et al. make an important contribution by identifying major gaps in public dementia prevention knowledge. Refining future assessments around actionable knowledge, risk-factor actionability, and psychologically distinct dimensions of risk perception may further strengthen public health strategies for dementia prevention.

## Financial support

None.

## Declarations of AI use

We have not used any AI at all.

## CRediT authorship contribution statement

**Zhiyan Xie:** Methodology, Writing – original draft, Writing – review & editing. **Jun Su:** Conceptualization, Writing – original draft. **Tao Liao:** Supervision, Writing – original draft, Writing – review & editing.

## Conflicts of interest

The authors declare that they have no known competing financial interests or personal relationships that could have appeared to influence the work reported in this paper.

## References

[1] Sambou M.L., Dobbe J.H.M., Albers E.A.C., Deckers K., Arends L.R., Ikram M.A. (2026). Knowledge and perception of dementia risk and protective factors: a systematic review and meta-analysis. J Prev Alzheimers Dis.

[2] Matton A., Stephen R., Daniilidou M., Barbera M., Alanko V., Ballin M. (2025). Mechanisms of interventions targeting modifiable factors for dementia risk reduction. Mol Neurodegener.

[3] Bransby L., Rosenich E., Maruff P., Lim Y.Y. (2024). How modifiable are modifiable dementia risk factors? A framework for considering the modifiability of dementia risk factors. J Prev Alzheimers Dis.

[4] Cavuoto M.G., Davies L., Rowsthorn E., Cribb L.G., Yiallourou S.R., Yassi N. (2024). Cross-sectional associations between neighborhood characteristics, cognition and dementia risk factor burden in middle-aged and older Australians. Prev Med Rep.

[5] Brigiano M., Calabrese L., Chirico I., Trolese S., Quartarone M., Forte L. (2025). Within my walls, I escape being underestimated: a systematic review and thematic synthesis of stigma and help-seeking in dementia. Behav Sci (Basel).

[6] Lin R., Bartels S.L., Batalik L., Su J.J. (2025). The moderating role of dementia-related fear in the relationship between perceived cognitive decline and motivation for dementia risk reduction behaviors in community-dwelling middle-aged and older adults. Int J Geriatr Psychiatry.

